# Infliximab is associated with an increased risk of serious infection in patients with psoriasis in the U.K. and Republic of Ireland: results from the British Association of Dermatologists Biologic Interventions Register (BADBIR)

**DOI:** 10.1111/bjd.17036

**Published:** 2018-10-21

**Authors:** Z.Z.N. Yiu, D.M. Ashcroft, I. Evans, K. McElhone, M. Lunt, C.H. Smith, S. Walton, R. Murphy, N.J. Reynolds, A.D. Ormerod, C.E.M. Griffiths, R.B. Warren, BADBIR Study Group

**Affiliations:** ^1^ Dermatology Centre Salford Royal NHS Foundation Trust Manchester M13 9PT U.K; ^2^ Centre for Pharmacoepidemiology and Drug Safety School of Health Sciences and Manchester M13 9PT U.K; ^3^ Arthritis Research U.K. Epidemiology Unit The University of Manchester Manchester Academic Health Science Centre NIHR Manchester Biomedical Research Centre Manchester M13 9PT U.K; ^4^ St. John's Institute of Dermatology Guy's and St Thomas’ NHS Foundation Trust London SE1 9RT U.K; ^5^ Department of Dermatology Castle Hill Hospital Hull HU16 5JQ U.K; ^6^ Sheffield University Teaching Hospitals and Sheffield Children's Hospitals Sheffield S10 2JF U.K; ^7^ Dermatological Sciences, Institute of Cellular Medicine Medical School, Newcastle University and Department of Dermatology Royal Victoria Infirmary Newcastle Hospitals NHS Foundation Trust Newcastle upon Tyne NE2 4HH U.K; ^8^ Division of Applied Medicine University of Aberdeen Aberdeen AB25 2ZD U.K

## Abstract

**Background:**

Patients with psoriasis and clinicians are concerned that infliximab may be associated with a risk of serious infections.

**Objectives:**

To compare the risk of serious infections associated with infliximab in patients with chronic plaque psoriasis against a cohort on nonbiologic systemic therapies.

**Methods:**

A prospective cohort study was performed using data from the British Association of Dermatologists Biologic Interventions Register (BADBIR). Infliximab was compared with nonbiologic systemic therapies, inclusive of any exposure to methotrexate, ciclosporin, acitretin, fumaric acid esters, psoralen‐ultraviolet A or hydroxycarbamide. Serious infections were those associated with hospitalization, the use of intravenous antimicrobial therapy and/or those that led to death. Propensity score inverse probability treatment weights were used to adjust for potential confounding from *a priori* identified covariates. Cox proportional hazards models were calculated to obtain hazard ratios (HRs).

**Results:**

In total, 3843 participants were included for analysis up to October 2016. The incidence rates were significantly higher in the infliximab cohort (47·8 per 1000 person‐years) [95% confidence interval (CI) 35·7–64·0], compared with 14·2 per 1000 person‐years (95% CI 11·5–17·4) in the nonbiologic systemic cohort. Infliximab was associated with an overall increase in the risk of serious infection compared with nonbiologics [adjusted HR (adjHR) 1·95, 95% CI 1·01–3·75] and methotrexate only (adjHR 2·96, 95% CI 1·58–5·57) and a higher risk of serious infection in the first 6 months of therapy (adjHR 3·49, 95% CI 1·14–10·70).

**Conclusions:**

Infliximab is associated with an increased risk of serious infections compared with nonbiologic systemic therapies in patients with psoriasis in the U.K. and the Republic of Ireland.

## Abstract 2

**What's already known about this topic?**

- Randomized clinical trials are not sufficiently powered to investigate the risk of serious infection in patients with psoriasis who are undergoing treatment with infliximab.
- Published observational studies have used different methods to adjust for confounding and different comparators.
- Previous studies also lacked the adequate sample size to obtain a precise estimate of the risk of serious infection for infliximab.

**What does this study add?**

- Using methods that better address bias and confounding, our study suggests that infliximab is associated with a higher risk of serious infections compared with nonbiologic systemic therapies in patients with psoriasis.
- Patients should be counselled on the risk of serious infection before infliximab is prescribed.

**Linked Comment:** Puig. *Br J Dermatol* 2019; **180**: 257–258.

Plain language summary available online

Respond to this article

Infliximab, a chimeric monoclonal antibody against tumour necrosis factor‐α, is highly efficacious for the treatment of psoriasis.^1^ In the U.K., infliximab use for psoriasis is reserved for patients with very severe disease, i.e. with a Psoriasis Area and Severity Index (PASI) ≥ 20 and Dermatology Life Quality Index (DLQI) > 18, compared with other biologic therapies where the disease severity criteria are lower, i.e. PASI ≥ 10, DLQI > 10. The British Association of Dermatologists guidelines for biologic therapies in psoriasis specifically recommend that infliximab is reserved for people with very severe disease or where other available biologic agents have failed or cannot be used.^2^ By indication, patients treated with infliximab are therefore substantially different to those treated with other biologic therapies.

One of the main adverse events leading to discontinuation of biologic therapies is infection.^3^ Serious infections, which are defined as those associated with significant morbidity or mortality, are therefore a legitimate concern for clinicians and patients. Most of the evidence for the quantification of the risk of serious infection in patients with psoriasis on infliximab is drawn from data in randomized clinical trials (RCTs) with important limitations including poor external validity,^4^ low event rates and unclear reporting of outcome.^5^ A systematic review of RCTs found that infliximab was associated with a nonstatistically significant increase in the risk of serious infection compared with placebo at weeks 20–30 [Peto odds ratio 3·53, 95% confidence interval (CI) 0·31–40·37].^5^ Two prospective observational cohort studies have reported on the risk of serious infection with infliximab in patients with psoriasis; one study with 184 patients (264·2 person‐years) on infliximab showed nonstatistically significant increased risk with infliximab compared with methotrexate,^6^ while another study showed statistically significant increased risk compared with retinoids/phototherapy in a prevalent cohort (1151 patients, 2253 person‐years) but not in an incident cohort (246 patients, 324 person‐years).^7^ Both observational studies were limited by a small incident cohort of patients on infliximab.

Our objective is to determine whether infliximab elevates the risk of serious infection above that of nonbiologic systemic therapies in patients with psoriasis, using a large, national, prospective psoriasis registry – the British Association of Dermatologists Biologic Interventions Register (BADBIR).

## Patients and methods

BADBIR is a national prospective ongoing pharmacovigilance registry of patients with psoriasis that was established in 2007 in the U.K. and Republic of Ireland to compare the safety of biologic therapies vs. nonbiologic systemic therapies. Establishing the risk of serious infections was a prespecified primary aim of the registry. The design of BADBIR^8^ and the baseline patient characteristics^9^ have been published previously. The National Institute for Health and Care Excellence (NICE) recommends that all patients with psoriasis on biologic therapies should be registered on BADBIR. Patients were selected using a data snapshot from October 2016. BADBIR was approved in March 2007 by the National Health Service Research Ethics Committee North West England (07/MRE08/9). All patients gave written informed consent for their participation in the registry.

### Baseline assessment

Baseline data were collected before or during the initial 6 months of treatment. Drug, clinical and comorbidity history along with anthropometric data were collected by a healthcare professional using a web‐administered questionnaire, whereas lifestyle factors were collected using a patient‐completed questionnaire.

### Follow‐up assessments

Data from patients were collected every 6 months for the first 3 years, then annually thereafter up to 10 years. Follow‐up data were collected and entered into a web‐based system contemporaneously. Specific information about serious infections were collected, including descriptions of events, hospitalization, start and stop dates. Adverse events were classified using the Medical Dictionary for Regulatory Activities (MedDRA) system.

### Data analysis

The main inclusion criteria for this study were patients with chronic plaque psoriasis starting infliximab (Remicade^®^, Johnson & Johnson, New Brunswick, NJ, U.S.A.) and biologic‐naïve patients with chronic plaque psoriasis on acitretin, psoralen‐ultraviolet A, ciclosporin, fumaric acid esters, methotrexate or hydroxycarbamide, who were recruited in the nonbiologic systemic cohort. Owing to difficulties such as a low sample size for infliximab and a lack of comparable patients receiving other biologic therapies, adalimumab, etanercept and ustekinumab were analysed separately.^10^ Analysis of patients who were biologic‐naïve (i.e. first‐line infliximab therapy) was performed separately to the aggregated analysis with all patients in the infliximab cohort (i.e. all‐lines infliximab therapy).

Patients were included if follow‐up data (at least one follow‐up) were available. Overall, 844 patients were not included; three emigrated, 785 withdrew consent and 56 did not complete their questionnaire. Patients on infliximab contributed follow‐up time from the first dose until the first occurrence of the following events: serious infection, discontinuation of treatment owing to other reasons, last registered follow‐up, switch to other biologic therapy or death. Patients in the nonbiologic cohort contributed follow‐up time from first dose of the index drug until the first event of any of the above, but were censored at the end of the last alternative nonbiologic therapy. Patients who switched from the nonbiologic therapy cohort to start infliximab contributed follow‐up time to both cohorts.

A serious infection was defined as any infection that was associated with or prolonged hospitalization, use of intravenous antimicrobial therapy or led to death. The events were validated by separate review from two clinicians (Z.Z.N.Y. and R.B.W.) against the above criteria, and discrepancies were resolved through discussion. A clinical specialist in the specific type of infection was consulted in cases where there was uncertainty. The first serious infection was included for analysis in the current study, with a risk window period of 90 days following cessation of treatment applied for the attribution of the event to the drug.^11^

The impact of alternate dosing regimens was not analysed as the proportion of patients using cumulative doses that differ from the licensed dosing regimens is low in the U.K. (< 15%)^12^ and the NICE‐approved dosing regimen is in accordance with the licence. Within the infliximab cohort, the number of person‐years receiving doses outside the licence was too low to make statistical inferences regarding the effect of dosing regimen on the risk of serious infection.

### Primary analyses

To provide a description of the rates of serious infections, crude incidence rates for each drug in the biologic cohort and in the nonbiologic cohort were calculated as the number of events per 1000 patient‐years of follow‐up. Survival modelling with Cox proportional hazards was used to compare event rates and estimate the effect of each exposure on the risk of serious infections. To investigate whether the risk of serious infections was time varying, we used the crude incidence rates at 0–6 months, 6–12 months and 12–24 months of follow‐up, which are the designated follow‐up data reporting time points.

The specific *a priori* potential confounders that were included in the multivariable analysis were based on expert opinion and a literature review.^5^ These were age, sex, body mass index (BMI), waist circumference, alcohol use, disease severity (PASI), concomitant inflammatory arthritis (including psoriatic arthritis and ankylosing spondylitis), smoking, diabetes, chronic obstructive pulmonary disease, asthma and concomitant immunosuppressants. The total number of measured comorbidities was included as a separate covariate as a proxy for patient frailty. BMI was presented as a categorical variable to ease data description in Table 1, but was kept as a continuous variable in the statistical models. Adjustment for the baseline potential confounders was performed using a propensity score model. A probability score for having the treatment was derived from a logistic regression model based on the baseline relevant covariates listed above. The use of propensity score adjustment has various advantages over multivariable regression models, in particular the ability to check the balance of measured confounders between the comparator cohorts, and improve estimation when an outcome is rare by allowing for multiple covariates.^13^

**Table 1:** The baseline demographic and disease characteristics of the infliximab and nonbiologic cohort

Characteristics	Nonbiologic cohort (*n* = 3421)	First‐line infliximab (*n* = 105)	All‐lines infliximab (*n* = 422)
Age (years), mean ± SD	44·6 ± 14·0	46·6 ± 13·5	46·6 ± 12·7
Female sex	1489 (43·5)	32 (30·5)	159 (37·7)
Waist circumference (cm), mean ± SD	99·7 ± 17·1	105·6 ± 19·6	106·1 ± 18·7
BMI category (kg m^−2^)			
Underweight (< 18·5)	43 (1·3)	1 (1·0)	2 (0·5)
Normal (18·5–24·9)	677 (19·8)	19 (18·1)	54 (12·8)
Overweight (25·0–29·9)	1071 (31·3)	18 (17·1)	93 (22·0)
Obese I (30·0–34·9)	735 (21·5)	20 (19·0)	86 (20·4)
Obese II (35·0–39·9)	345 (10·1)	15 (14·3)	60 (14·2)
Obese III (≥ 40)	279 (8·2)	21 (20·0)	64 (15·2)
Comorbidities and risk factors			
No comorbidity	1323 (38·7)	25 (23·8)	98 (23·2)
1–2 comorbidities	1585 (46·3)	48 (45·7)	197 (46·7)
3–4 comorbidities	416 (12·2)	23 (21·9)	92 (21·8)
≥ 5 comorbidities	97 (2·8)	9 (8·6)	35 (8·3)
Hypertension	620 (18·1)	35 (33·3)	142 (33·6)
Past TB	21 (0·6)	2 (1·9)	9 (2·1)
Diabetes mellitus	254 (7·4)	15 (14·3)	56 (13·3)
Dyslipidaemia	307 (9·0)	14 (13·3)	63 (14·9)
Asthma	361 (10·6)	9 (8·6)	54 (12·8)
COPD	69 (2·0)	1 (1·0)	7 (1·7)
Number of cigarettes smoked per day, mean ± SD	4·6 ± 7·7	5·9 ± 8·8	4·8 ± 9·5
Alcohol units per week, mean ± SD	7·7 ± 12·1	10·9 ± 23·8	8·1 ±16·0
Disease			
Disease duration (years), median (IQR)	18·0 (18·0)	19·0 (16·0)	20·6 (17·2)
Baseline PASI score, median (IQR)	14·1 (7·9)	24·6 (12·3)	20·3 (13·7)
Inflammatory arthritis	363 (10·6)	33 (31·4)	164 (38·9)
Treatment history			
First‐line biologic/nonbiologic therapy	3421 (100·0)	105 (100·0)	105 (24·9)
Second‐line biologic therapy			131 (31·0)
Third‐line biologic therapy			123 (29·1)
Fourth‐line (or more) biologic therapy			63 (14·9)
Concomitant treatments			
Any exposure to methotrexate during follow‐up	2118 (61·9)	28 (26·7)	148 (35·1)
Any exposure to ciclosporin during follow‐up	1216 (35·6)	6 (5·7)	43 (10·2)
Any exposure to fumaric acid esters during follow‐up	552 (16·1)	2 (1·9)	7 (1·7)
Any exposure to hydroxycarbamide during follow‐up	56 (1·6)	5 (4·8)	9 (2·1)

BMI, body mass index; PASI, Psoriasis Area and Severity Index. List of predefined comorbidities included hypertension, angina, myocardial infarction, stroke, epilepsy, asthma, chronic obstructive pulmonary disease (COPD), peptic ulcer disease, chronic renal disease, liver disease, previous tuberculosis (TB), demyelination, diabetes mellitus, impaired glucose tolerance, depression, dyslipidaemia, nonskin cancer, immunodeficiency syndromes and thyroid disease. Data are provided as *n* (%) unless otherwise stated.

Inverse probability treatment weighting, where the treatments were weighted for the distribution of the propensity score in the whole model cohort, was then performed using propensity score probabilities in both models. Balance between groups after weighting was assessed using expected bias from a logistic regression model estimating the effect of the variable on serious infection. Improvement in balance was achieved by an iterative process of fitting interaction terms involving the least balanced variables.

Concomitant therapies considered to be immunosuppressants were methotrexate, ciclosporin, fumaric acid esters and hydroxycarbamide. Concomitant immunosuppressants (defined as the exposure period to more than one immunosuppressant in the nonbiologic cohort) were treated exceptionally as time‐varying covariates, allowing for the time on and off these drugs throughout follow‐up.

Missing data (Table S1; see Supporting Information) were imputed in a multiple imputation model of 20 datasets in order to reduce bias.^14^ We used multiple imputations to account for missing data for the potential confounders, as this preserves the variability and uncertainty of the missing data and avoids loss of power and bias that alternative ad hoc methods, such as a complete case analysis, may introduce. Propensity likelihood scores were calculated in each imputed dataset and combined after regression modelling using Rubin's rules. A key assumption for the Cox regression is the proportionality assumption, where the relative risk between the comparators is constant over time. Formal testing for proportionality using Schoenfeld's residuals in the Cox regression model was performed in five extracted imputed datasets, and, where the proportionality assumption did not hold, a time‐stratified analysis using the prespecified time points was performed.

### Secondary analyses

*A priori* planned sensitivity analysis included methotrexate users as the comparator cohort as this was the most common systemic nonbiologic in use. Descriptive analysis was performed for soft tissue and skin infections and lower respiratory tract infections as these were identified as common infections associated with patients on biologic therapies, but the lower number of events did not allow for meaningful multivariable analysis of relative risks (Table S2; see Supporting Information).

All analyses were performed using Stata 14 (StataCorp LLC, College Station, TX, U.S.A.). The methods used in this analysis have been previously described.^10^ The details of the methodology pertaining to this current study are reproduced in full for the benefit of the reader.

## Results

In total, 3843 participants were included in the analysis, with 3421 participants included in the nonbiologic systemic cohort and 422 participants included in the all‐lines infliximab cohort up to October 2016. Overall, 105 biologic‐naïve participants were started on first‐line infliximab therapy. The baseline demographic, anthropometric and disease characteristics of the participants are listed in Table 1. The total and median follow‐up time for all lines of infliximab was 941·1 person‐years and 1·49 person‐years [interquartile range (IQR) 2·50 person‐years], respectively. For biologic‐naïve patients on infliximab the total follow‐up time was 238·87 and the median follow‐up time was 1·84 person‐years (IQR 2·70 person‐years) and for the nonbiologic cohort the total and median follow‐up time was 6419·24 person‐years and 1·51 person‐years (IQR 1·84 person‐years), respectively.

### Crude incidence rates for serious infections overall

The incidence rate for serious infections in the nonbiologic cohort was 14·18 per 1000 person‐years (95% CI 11·54–17·41), with the incidence rate for the methotrexate only cohort at 11·98 per 1000 person‐years (95% CI 8·82–16·27). The crude incidence rate in the entire infliximab cohort was 47·82 per 1000 person‐years (95% CI 35·70–64·04), and 58·61 per 1000 person‐years (95% CI 34·71–98·96) for the biologic‐naïve infliximab cohort.

The most common serious infections coded using MedDRA high level terms that were experienced by participants on either nonbiologic systemic therapy or infliximab were lower respiratory tract infections, followed by skin and soft tissue infections and urinary tract infections (Table S2; see Supporting Information). The crude incidence rates for lower respiratory tract infections and skin and soft tissue infections were higher for infliximab (Table 2). The median hospital inpatient stay was 3 days (IQR 6·0) for nonbiologic therapies and 2 days for infliximab (IQR 9·0).

**Table 2:** Crude incidence rates of first serious infection overall (lower respiratory tract infections; skin and soft tissue infections)

Treatment	*n*	Total person‐time (median follow‐up time), years	Infections	Rate (per 1000 person‐years)	95% CI (person‐years)
All serious infections					
Nonbiologics	3421	6419·24 (1·51)	91	14·18	11·54–17·41
Methotrexate	2118	3422·40 (1·27)	41	11·98	8·82–16·27
Infliximab (first line)	105	238·87 (1·84)	14	58·61	34·71–98·96
Infliximab (all lines)	422	935·22 (1·49)	45	47·82	35·70–64·04
Lower respiratory tract infections					
Nonbiologics			27	4·21	2·88–6·13
Methotrexate			14	4·09	2·42–6·91
Infliximab (first line)	105		<5	4·19	0·59–29·72
Infliximab (all lines)	422		11	11·69	6·47–21·11
Skin and soft tissue infections					
Nonbiologics			22	3·43	2·26–5·20
Methotrexate			10	2·92	1·57–5·43
Infliximab (first line)	105		5	20·93	8·71–50·29
Infliximab (all lines)	422		13	13·81	8·02–23·79

CI, confidence interval.

### Propensity score weighted models for the risk of serious infections

**Figure 1:**
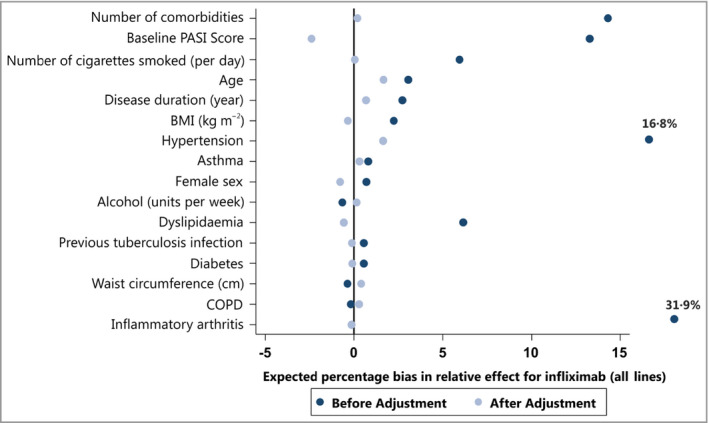
Forest plot showing the reduction in expected percentage bias for the individual covariates after inverse probability treatment weighted propensity score weighting. PASI, Psoriasis Area and Severity Index; BMI, body mass index; COPD, chronic obstructive pulmonary disease.

The inverse probability treatment weighted (IPTW) logistic regression model for infliximab vs. nonbiologic therapies achieved good balance, removing expected bias for most of the variables (Fig. 1 and Table S3; see Supporting Information), which suggested a reduction of confounding from these variables.

Infliximab showed a statistically significant increase in the risk of serious infection compared with nonbiologic systemic therapies overall [adjusted hazard ratio (HR) 1·95, 95% CI 1·01–3·75; Table 3]. The proportionality assumption was not met for this model, and therefore a split in a priori defined follow‐up time was performed. The risk of serious infection was significantly higher in the first 6 months (adjusted HR 3·49, 95% CI 1·14–10·70) and between 6 months and 1 year (adjusted HR 2·99, 95% CI 1·10–8·14) but not significantly higher between 1 and 2 years (adjusted HR 2·03, 95% CI 0·61–6·79) compared with the nonbiologic cohort (Table 3). The adjusted estimate for biologic‐naïve participants on infliximab was also higher than nonbiologic therapies but this was not statistically significant (adjusted HR 1·37, 95% CI 0·50–3·74).

**Table 3:** Crude and adjusted Cox proportional hazards models using inverse probability treatment weighting by propensity score. Exposure time with concomitant (methotrexate, ciclosporin, fumaric acid esters, hydroxycarbamide) immunosuppressive medication use is adjusted for, with exposure time to two immunosuppressive therapies classed as concomitant in the nonbiologic cohort

	Infliximab (all lines; *N* = 422) against nonbiologic therapies (95% CI)	*P*‐values	Infliximab (first line; *N* = 105) against nonbiologic therapies (95% CI)	*P*‐values	Infliximab (all lines) against methotrexate (95% CI)	*P*‐values	Infliximab (first line) against methotrexate (95% CI)	*P*‐values
Crude hazard ratio (HR) overall time (95% confidence interval)	3·41 (2·38–4·90)	< 0·001	4·19 (2·42–7·26)	< 0·001	4·39 (2·85–6·75)	< 0·001	5·16 (2·82–9·46)	< 0·001
Adjusted HR Overall	1·95 (1·01–3·75)	0·046	1·37 (0·50–3·74)	0·541	2·96 (1·58–5·57)	0·001	1·97 (0·71–5·46)	0·193
HR for concomitant immunosuppressant therapy	1·34 (0·61–2·97)	0·464	3·06 (1·41–6·67)	0·005	1·24 (0·59–2·60)	0·577	2·45 (1·02–5·88)	0·045
Crude HR for time 0–6 months	4·73 (2·07–10·81)	< 0·001						
Adjusted HR for time 0–6 months	3·49 (1·14–10·70)	0·029						
Crude HR for time 6–12 months	6·99 (3·63–13·45)	< 0·001						
Adjusted HR for time 6–12 months	2·99 (1·10–8·14)	0·032						
Crude HR for time 1–2 years	2·27 (1·08–4·76)	0·030						
Adjusted HR for time 1–2 years	2·03 (0·61–6·79)	0·249						

### Sensitivity analysis

Infliximab (all lines) had a statistically significant increase in the risk of serious infection compared with methotrexate (adjusted HR 2·96, 95% CI 1·58–5·57; Table 3). The proportionality assumption was met for this alternative model.

## Discussion

Infliximab was associated with a twofold increase in the risk of serious infections compared with nonbiologic systemic therapies, and a threefold increase in the risk of serious infections compared with methotrexate, in patients with psoriasis. This is in contrast to our results from other biologic therapies in BADBIR, where etanercept, adalimumab and ustekinumab were not associated with a higher risk of serious infections than nonbiologic systemic therapies. There was a time‐stratified difference in the risk of serious infections in the comparison between infliximab and nonbiologic therapies, with a higher risk in the first 6 months of therapy and a lower risk after 1 year of therapy, but these estimates had overlapping CIs given the fact that we had a lower precision owing to a lack of power within the specific time strata.

We have shown that combination treatment of infliximab with methotrexate, and other immunosuppressive therapies, is associated with a threefold increase in the risk of serious infection compared with patients on infliximab monotherapy in the biologic‐naïve cohort. This is not the case in the overall cohort (i.e. both biologic‐naïve and experienced) (Table 3). However, the propensity score method balances the baseline characteristics and not time‐varying factors, and hence it cannot deal adequately with confounding by indication for the use of concomitant immunosuppressive therapy. Therefore, this estimated result should be interpreted with caution.

The major strengths of this study are the prospective cohort study design, fully industry‐independent data analysis and the participation of multiple dermatology centres (*N* = 153) in the U.K. and Republic of Ireland. This study reports on the risk of serious infection in the largest incident cohort of patients with psoriasis on infliximab to date. Owing to the capture of numerous important covariates, we were able to account for significant confounding through weighting by propensity score. The substantial reduction in the point estimate of the HR after adjustment (> 40% change) suggests that significant positive confounding was reduced after IPTW propensity score adjustment.

Limitations include recall bias, which may occur with patient‐reported characteristics, and residual confounding through variables that were not measured and not known to be associated with either exposure or outcome. There is a possibility that clinicians may have a lower threshold and a heightened awareness for admitting patients on infliximab with suspected infections compared with nonbiologic systemic therapies, thereby introducing confounding by indication that cannot be adjusted for. As patients on infliximab are by indication (higher PASI and DLQI) different from all other patients on the registry, there may be a higher degree of selection bias in our comparisons. The potential confounding introduced by this selection bias may be partly adjusted for from the measured covariates, and the propensity score IPTW method reduced expected bias substantially (Table S3; see Supporting Information), but there may be unmeasured covariates that have introduced confounding that cannot be adjusted for. For example, we were not able to adjust for previous serious infection within the past year. Conversely, our results have high external validity for patients with psoriasis who are eligible for infliximab in the U.K. or any other country with similar prescription eligibility criteria for the drug. A total of 844 registered patients were classified as dropping out owing to lack of follow‐up. Of these patients, 92·5% (785 patients) discontinued the study owing to withdrawal of consent and, because the reason for withdrawal is not provided, it is unclear how or whether this would have introduced systematic bias.

We performed an analysis with an incident cohort, which avoids left truncation. Left truncation occurs when there is a period of time during which the event could have occurred that cannot be observed. Involving a prevalent cohort would introduce left truncation by selecting the involvement of patients on infliximab who have not discontinued owing to a serious infection, and selecting out those patients who have discontinued infliximab owing to a serious infection, thereby underestimating the associated risk in infliximab. To maximize our sample size we included all lines of infliximab therapy (i.e. biologic‐experienced individuals who progressed onto infliximab as the second‐line, third‐line and fourth‐line biologic therapy); however, we also performed a sensitivity analysis restricted to first‐line infliximab therapy in biologic‐naïve participants. There is a possibility that patients with severe psoriasis who experienced a serious infection on the first‐line biologic therapy are not subsequently prescribed infliximab. However, it is reassuring that the results are contrary to this hypothesis, as the adjusted results for infliximab involving all lines of therapy are higher than those of first‐line therapy with tighter CIs, suggesting that the difference is due to sample size and power rather than left truncation.

Our crude incidence rate for serious infections for infliximab (47·8 per 1000 person‐years) is higher than that published in the Psoriasis Longitudinal Assessment and Registry (PSOLAR), a large study based mainly in the U.S.A. and Europe sponsored by a single pharmaceutical company (infliximab serious infection rate of 24·9 per 1000 person‐years),^7^ and the Spanish Registry of Adverse Events from Biological Therapy in Psoriasis (BIOBADADERM) (infliximab serious infection rate of 18·9 per 1000 person‐years).^6^ However, the adjusted relative risks from these cohorts are broadly similar to our results. PSOLAR reported an adjusted HR of 2·51 (95% CI 1·45–4·33) in a mixed prevalent/incident population and an adjusted HR of 1·78 in the incident population (95% CI 0·64–4·98), where the chosen comparator was a cohort on acitretin and/or phototherapy. BIOBADADERM reported an adjusted rate ratio of 2·52 (95% CI 0·83–7·69) in an incident cohort where the chosen comparator was a cohort on methotrexate only. The use of prevalent cohorts should be avoided in the assessment of adverse events because of the risk of left truncation, especially given the finding of an early high risk of serious infections in the first 90 days of treatment for infliximab in rheumatoid arthritis.^11^ Both PSOLAR and BIOBADADERM incident cohorts did not have the requisite power to achieve adequate estimate precision.

As discussed previously, infliximab is currently recommended for patients with severe psoriasis only.^2^ A recent network meta‐analysis of evidence from clinical trials has found infliximab to be of high efficacy but of poorer tolerability.^1^ Given our findings of a higher risk of serious infection associated with infliximab, we provide real‐world evidence to reinforce the position of infliximab in the psoriasis treatment hierarchy. However, it should be noted that there is no real‐world evidence, as yet, for the risk of serious infection of alternative, newer, licensed biologic therapies for psoriasis, such as secukinumab or ixekizumab.

Infliximab is associated with an overall twofold increase in the risk of serious infections when compared with nonbiologic systemic therapies. Patients with severe psoriasis who fulfil the criteria for the prescription of infliximab should be counselled for the risk of serious infection. These results are relevant to patients in the U.K. and the Republic of Ireland, and also in countries that have similar eligibility criteria for the prescription of infliximab.

## Conflicts of interest

A.D.O. has received consultation fees from Janssen, Eli Lilly, Amgen and Leo, speaker fees from Novartis and unrestricted research support from Merck, Pfizer, Janssen and AbbVie.

C.E.M.G. is a National Institute for Health Research Senior Investigator. C.E.M.G. reports grants from National Institute for Health Research during the conduct of the study, grants and personal fees from GlaxoSmithKline, AbbVie, Lilly, Novartis, Pfizer, Janssen, Leo, Celgene, grants from Sandoz, personal fees from Sun Pharmaceuticals, UCB Pharma, Almirall, in addition to grants and personal fees from outside the submitted work. C.H.S. has received research grants from AbbVie, Pfizer, Novartis, GlaxoSmithKline, Roche and Regeneron. D.M.A. was supported by a research grant from AbbVie and has received personal fees from Pfizer and GlaxoSmithKline. N.J.R. reports grants from PSORT industrial partners as listed in manuscript during the conduct of the study, other research grants from AstraZeneca and Stiefel GlaxoSmithKline, and other income to Newcastle University from Almiral, Amgen, Janssen and Novartis for lectures/attendance at advisory boards. R.B.W. has received research grants from AbbVie, Pfizer, Novartis and Leo, and reports personal fees from AbbVie, Amgen, Boehringer Ingelheim Pharma, Celgene, Janssen‐Cilag, Leo, Lilly, Novartis, Pfizer and Xenoport outside the submitted work. S.W. reports personal fees from AbbVie and Novartis outside the submitted work. Z.Z.N.Y. has received nonfinancial support from Novartis outside the submitted work.

## Supporting information


**Table S1** Missing data.
**Table S2** Location type of common first serious infections as coded by the Medical Dictionary for Regulatory Activities (MedDRA) high level terms (HLTs); percentage proportion of type of serious infection out of participants on each drug.
**Table S3** The baseline characteristics/variables after inverse probability treatment weighting by propensity score for treatment with infliximab.

**Powerpoint S1** Journal Club Slide Set.

**Video S1** Author video.

## Funding sources

British Association of Dermatologists Biologic Interventions Register (BADBIR) is coordinated by the University of Manchester and funded by the British Association of Dermatologists (BAD). The BAD receives income from Pfizer, Janssen Cilag, AbbVie, Novartis, Samsung Bioepis and Eli Lilly for providing pharmacovigilance services. This income finances a separate contract between the BAD and the University of Manchester who coordinate BADBIR. All decisions concerning analysis, interpretation and publication are made independently of any industry contribution. All relevant information regarding serious adverse events mentioned in this publication has been reported to the appropriate company as per the contractual agreements/standard operating procedures. Z.Z.N.Y. is funded by a National Institute for Health Research (NIHR) Doctoral Research Fellowship (DRF‐2015‐08‐089). BADBIR acknowledges the support of the NIHR through the clinical research networks and the contribution made by clinical research associates who continue to facilitate recruitment into the registry. The research was supported by the NIHR Manchester, Newcastle and the Guy's and St Thomas’ Biomedical Research Centres. The views expressed are those of the authors and not necessarily those of the National Health Service, the NIHR or the Department of Health. C.E.M.G. is an NIHR Senior Investigator.

A.D.O., C.E.M.G., C.H.S., D.M.A., N.J.R. and R.B.W. are funded, in part, by the Medical Research Council (MRC) (MR/L011808/1). N.J.R. is also funded by the MRC/Engineering and Physical Sciences Research Council Molecular Pathology Node.
