# Supplementary material for: Infliximab is associated with an increased risk of serious infection in patients with psoriasis in the U.K. and Republic of Ireland: results from the British Association of Dermatologists Biologic Interventions Register (BADBIR)
Source: Br J Dermatol. 2018 Oct 21;180(2):329–37. doi: 10.1111/bjd.17036 (PMC7379582; doi:10.1111/bjd.17036)
Supplement: Supplementary file 1 — Table S1 Missing data. Table S2 Location type of common first serious infections as coded by the Medical Dictionary for Regulatory Activities (MedDRA) high level terms (HLTs); percentage proportion of type of serious infection out of participants on each drug. Table S3 The baseline characteristics/variables after inverse probability treatment weighting by propensity score for treatment with infliximab. [file BJD-180-329-s001.docx]

**Supplementary Table S1 - Missing data**

| **Variable** | **N** | **%** |
| --- | --- | --- |
| **Disease duration** | 44 | 1.1% |
| **BMI** | 357 | 9.3% |
| **Alcohol** | 479 | 12.5% |
| **Waist** | 571 | 14.9% |
| **Smoking** | 827 | 21.5% |
| **PASI** | 1224 | 31.9% |

**Supplementary Table S2 - Location type of common first serious infections as coded by the MEDDRA High Level Terms (HLT); percentage proportion of type of serious infection out of participants on each drug**

| **MEDDRA HLT Codes** | **Non-biologics** | **Infliximab** |
| --- | --- | --- |
| Lower respiratory tract infections and lung infections | 27 (0.8%) | 11 (2.6%) |
| Skin structures and soft tissue infections | 22 (0.7%) | 13 (3.1%) |
| Urinary tract infections | 8 (0.2%) | <5 |
| Abdominal and gastrointestinal infections | 8 (0.2%) | <5 |
| Upper respiratory tract infections | 7 (0.2%) | <5 |
| Sepsis, bacteraemia, viraemia and fungaemia | <5 | <5 |
| Bone and joint infections | <5 | <5 |

**Supplementary Table S3 - The baseline characteristics/variables after inverse probability treatment weighting by propensity score for treatment with infliximab.**

| **Characteristic** | **Non-biologics** | **Infliximab** | **Expected bias in β before weighting** | **Expected bias in β after weighting** |
| --- | --- | --- | --- | --- |
| Age | 44.80 | 46.03 | 3.0% | 1.8% |
| Waist | 100.18 | 98.35 | -0.2% | 0.1% |
| BMI | 30.37 | 29.94 | 1.8% | -0.3% |
| Alcohol (units per week) | 7.84 | 8.79 | -0.5% | -0.5% |
| PASI | 16.54 | 15.59 | 12.2% | -2.2% |
| Disease duration | 19.56 | 20.76 | 3.1% | 1.0% |
| Smoking (no. cigarettes per day) | 5.10 | 5.12 | 7.4% | -0.7% |
| Number of comorbidities | 1.26 | 1.27 | 14.3% | 1.3% |
| Female gender | 43.0% | 49.0% | 0.6% | 0.5% |
| Hypertension | 20.0% | 21.0% | 16.8% | 0.2% |
| Asthma | 11.0% | 12.0% | 0.8% | -0.1% |
| COPD | 2.0% | 2.0% | -0.2% | -0.1% |
| Previous TB | 1.0% | 1.0% | 0.5% | -0.7% |
| Diabetes | 8.0% | 7.0% | 0.4% | -0.0% |
| Dyslipidaemia | 10.0% | 9.0% | 6.1% | 0.0% |
| Inflammatory arthritis | 14.0% | 14.0% | 31.9% | 0.3% |
