# Supplementary material for: Infliximab is associated with an increased risk of serious infection in patients with psoriasis in the U.K. and Republic of Ireland: results from the British Association of Dermatologists Biologic Interventions Register (BADBIR)
Source: Br J Dermatol. 2018 Oct 21;180(2):329–37. doi: 10.1111/bjd.17036 (PMC7379582; doi:10.1111/bjd.17036)
Supplement: Supplementary file 2 — Powerpoint S1 Journal Club Slide Set. [file BJD-180-329-s002.pptx]

## Slide 1
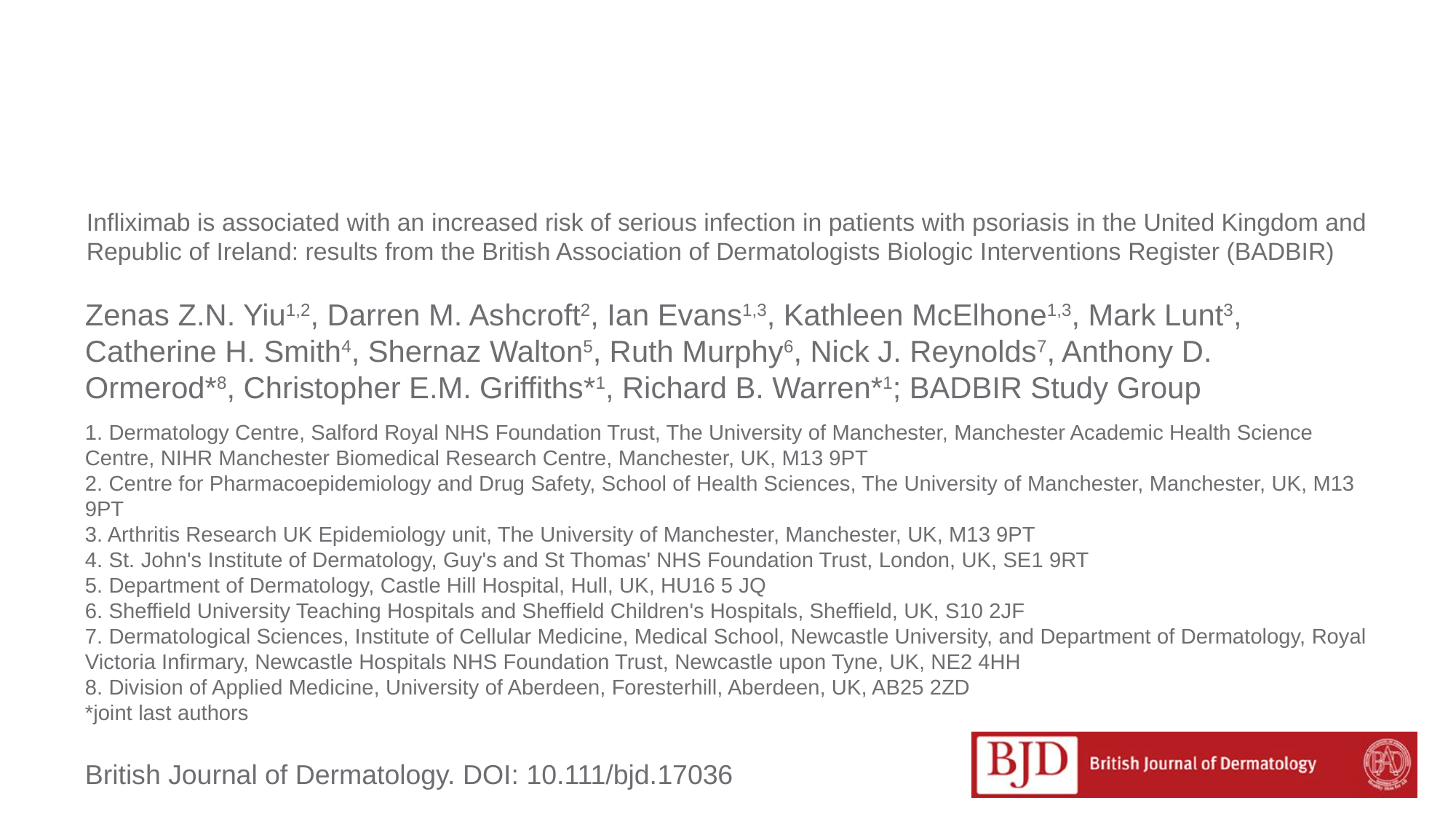

# Infliximab is associated with an increased risk of serious infection in patients with psoriasis in the United Kingdom and Republic of Ireland: results from the British Association of Dermatologists Biologic Interventions Register (BADBIR)
Zenas Z.N. Yiu1,2, Darren M. Ashcroft2, Ian Evans1,3, Kathleen McElhone1,3, Mark Lunt3, Catherine H. Smith4, Shernaz Walton5, Ruth Murphy6, Nick J. Reynolds7, Anthony D. Ormerod*8, Christopher E.M. Griffiths*1, Richard B. Warren*1; BADBIR Study Group
1. Dermatology Centre, Salford Royal NHS Foundation Trust, The University of Manchester, Manchester Academic Health Science Centre, NIHR Manchester Biomedical Research Centre, Manchester, UK, M13 9PT
2. Centre for Pharmacoepidemiology and Drug Safety, School of Health Sciences, The University of Manchester, Manchester, UK, M13 9PT
3. Arthritis Research UK Epidemiology unit, The University of Manchester, Manchester, UK, M13 9PT
4. St. John's Institute of Dermatology, Guy's and St Thomas' NHS Foundation Trust, London, UK, SE1 9RT
5. Department of Dermatology, Castle Hill Hospital, Hull, UK, HU16 5 JQ
6. Sheffield University Teaching Hospitals and Sheffield Children's Hospitals, Sheffield, UK, S10 2JF
7. Dermatological Sciences, Institute of Cellular Medicine, Medical School, Newcastle University, and Department of Dermatology, Royal Victoria Infirmary, Newcastle Hospitals NHS Foundation Trust, Newcastle upon Tyne, UK, NE2 4HH
8. Division of Applied Medicine, University of Aberdeen, Foresterhill, Aberdeen, UK, AB25 2ZD
*joint last authors
British Journal of Dermatology. DOI: 10.111/bjd.17036

## Slide 2
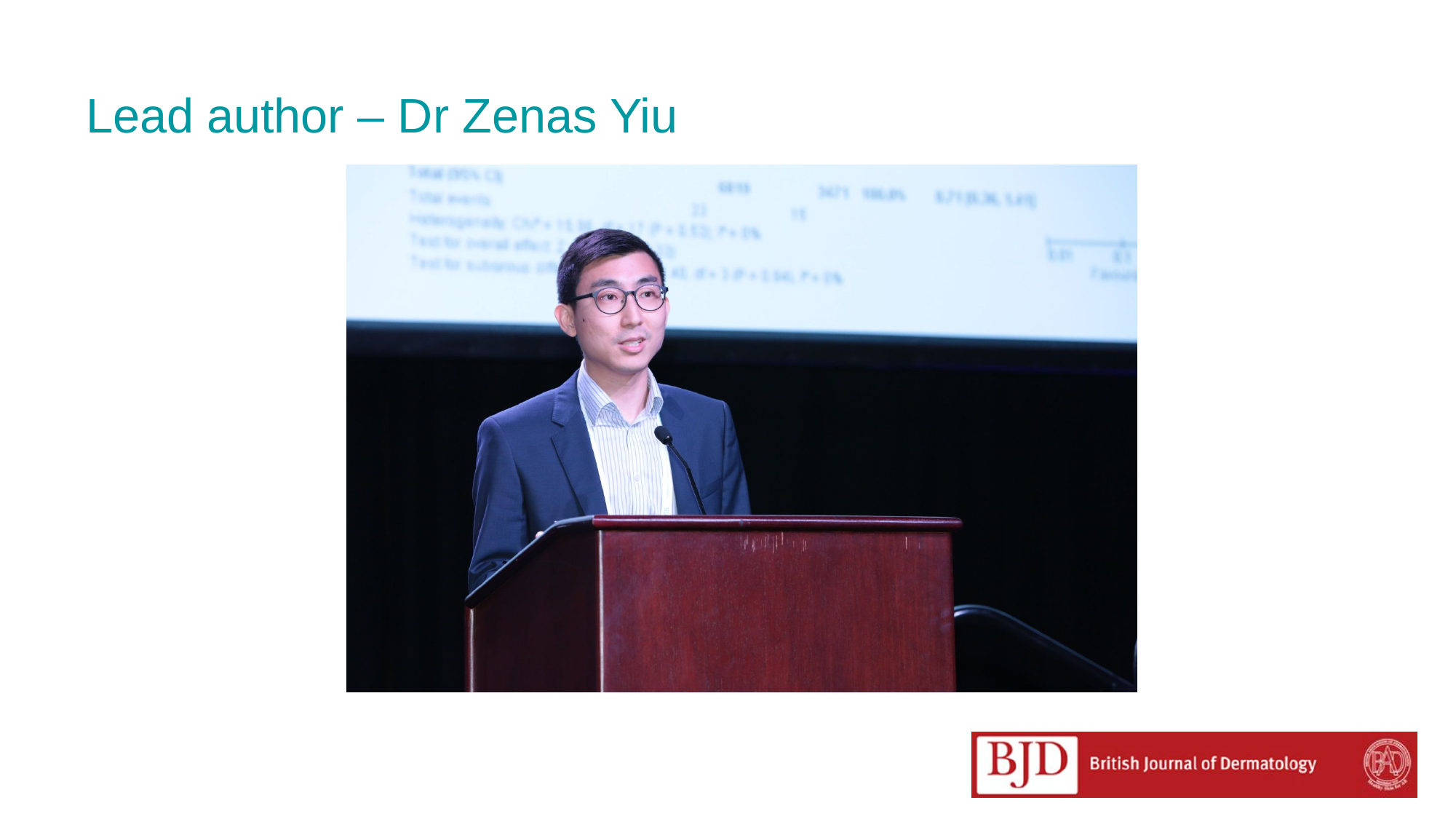

# Lead author – Dr Zenas Yiu

## Slide 3
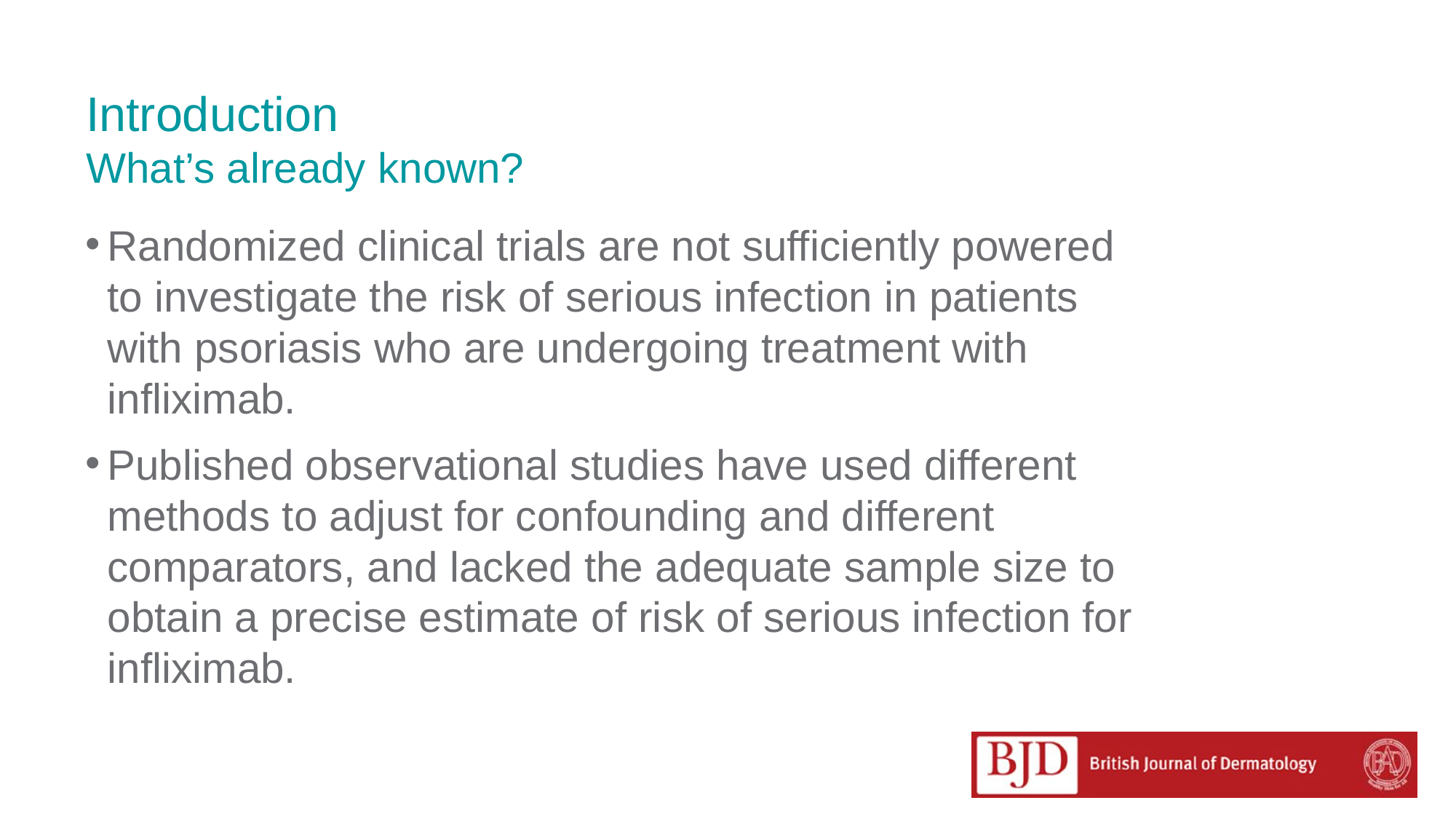

# Introduction What’s already known?
Randomized clinical trials are not sufficiently powered to investigate the risk of serious infection in patients with psoriasis who are undergoing treatment with infliximab.
Published observational studies have used different methods to adjust for confounding and different comparators, and lacked the adequate sample size to obtain a precise estimate of risk of serious infection for infliximab.

## Slide 4
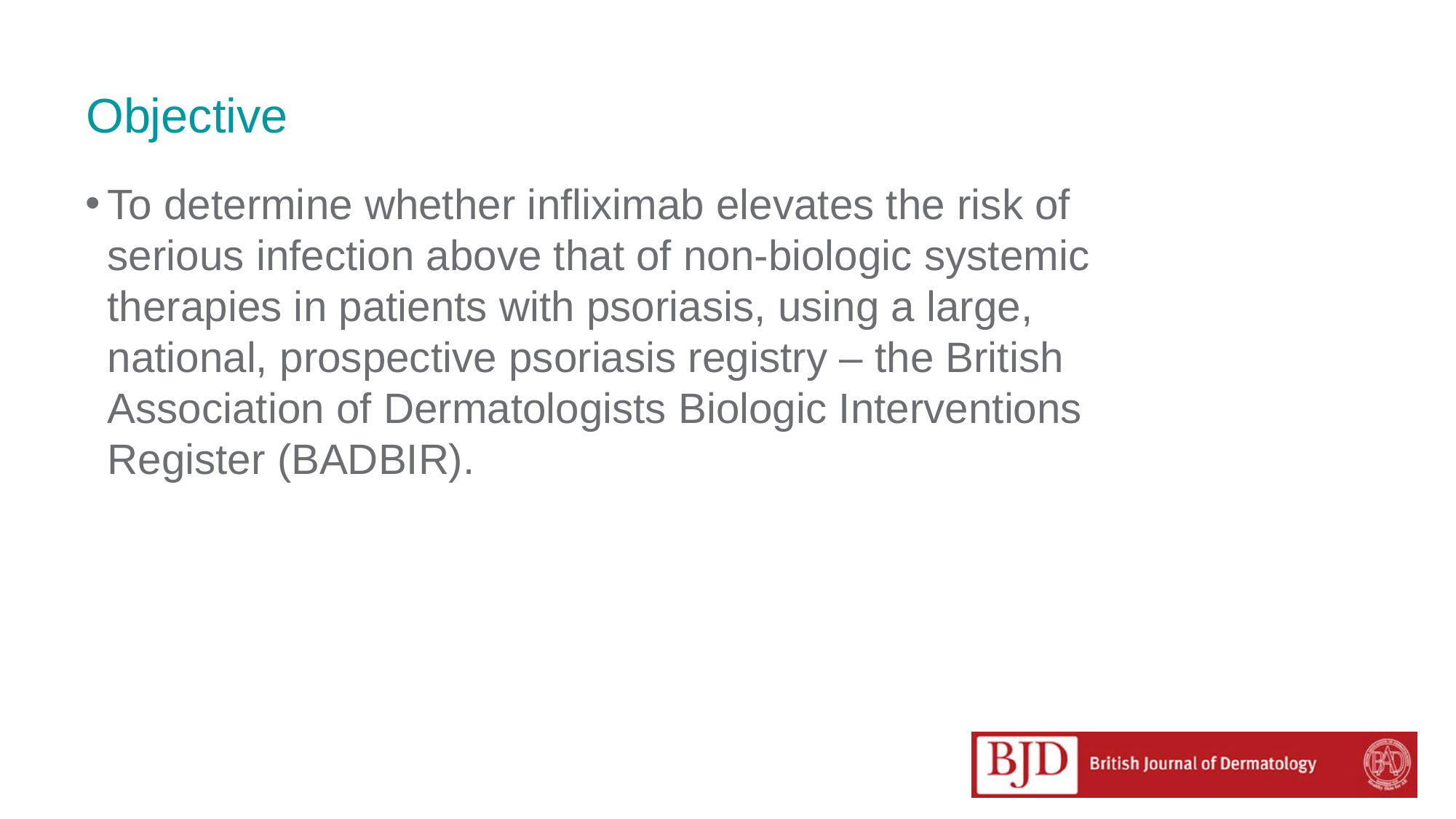

# Objective
To determine whether infliximab elevates the risk of serious infection above that of non-biologic systemic therapies in patients with psoriasis, using a large, national, prospective psoriasis registry – the British Association of Dermatologists Biologic Interventions Register (BADBIR).

## Slide 5
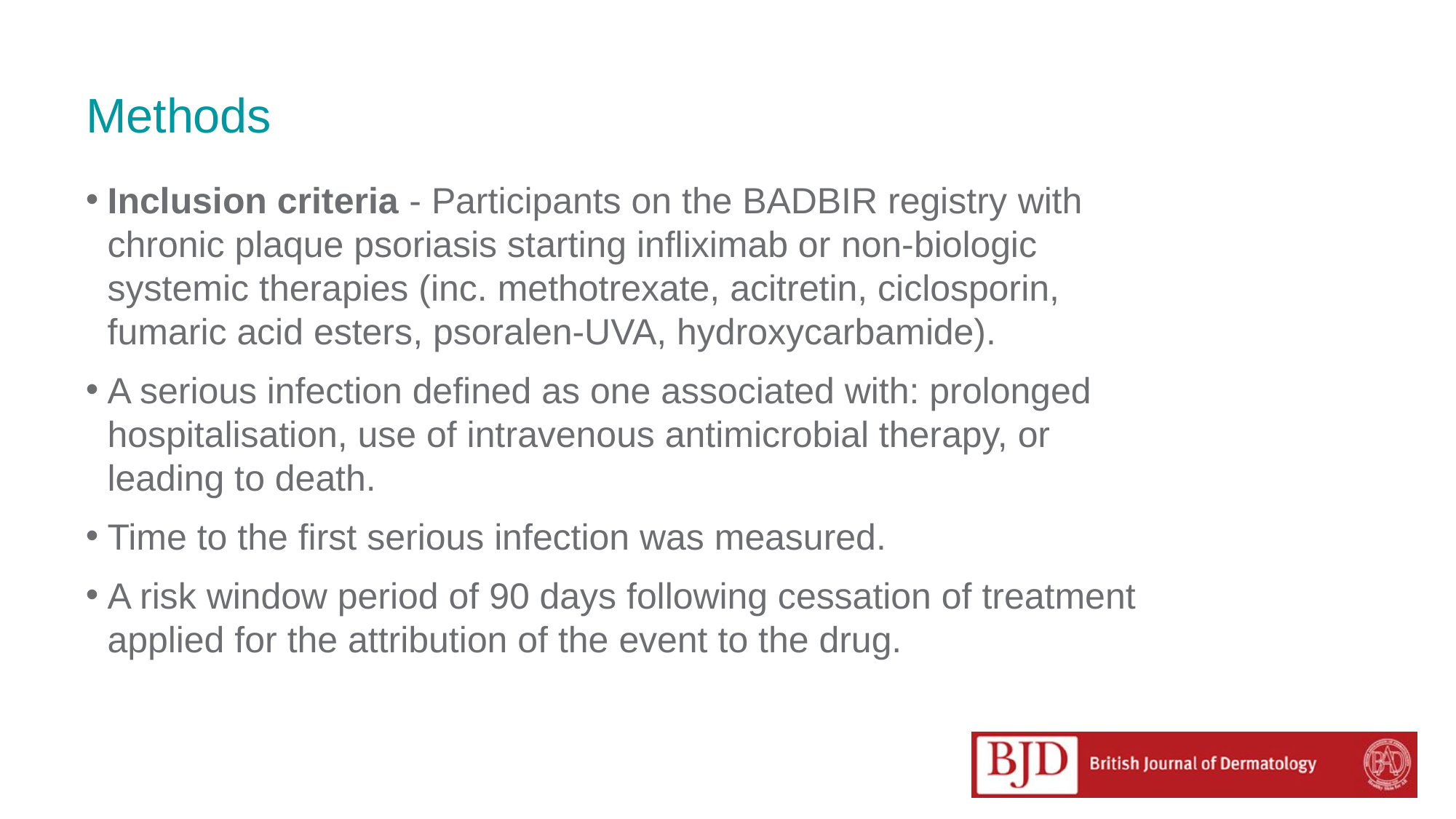

# Methods
Inclusion criteria - Participants on the BADBIR registry with chronic plaque psoriasis starting infliximab or non-biologic systemic therapies (inc. methotrexate, acitretin, ciclosporin, fumaric acid esters, psoralen-UVA, hydroxycarbamide).
A serious infection defined as one associated with: prolonged hospitalisation, use of intravenous antimicrobial therapy, or leading to death.
Time to the first serious infection was measured.
A risk window period of 90 days following cessation of treatment applied for the attribution of the event to the drug.

## Slide 6
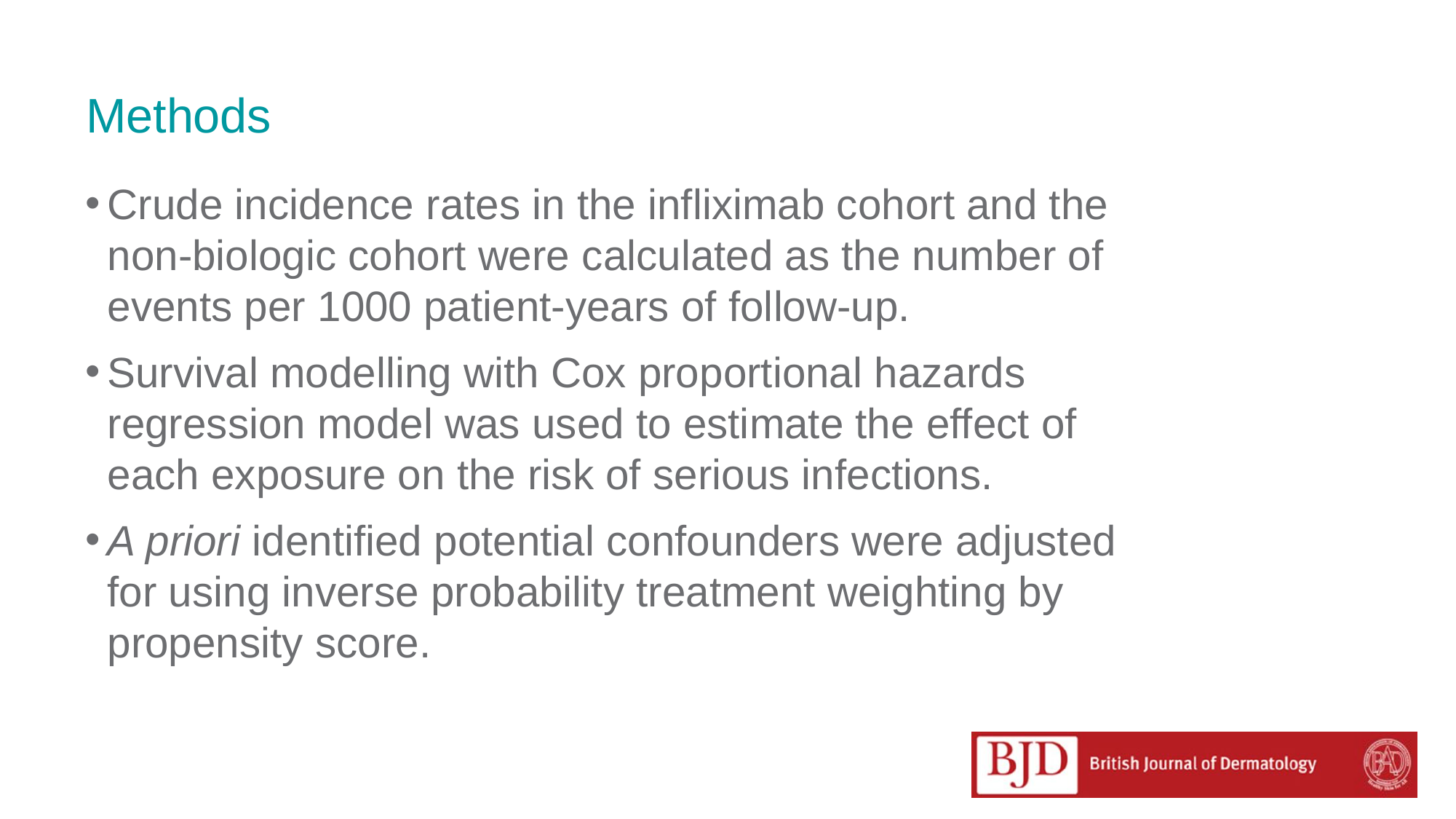

# Methods
Crude incidence rates in the infliximab cohort and the non-biologic cohort were calculated as the number of events per 1000 patient-years of follow-up.
Survival modelling with Cox proportional hazards regression model was used to estimate the effect of each exposure on the risk of serious infections.
A priori identified potential confounders were adjusted for using inverse probability treatment weighting by propensity score.

## Slide 7
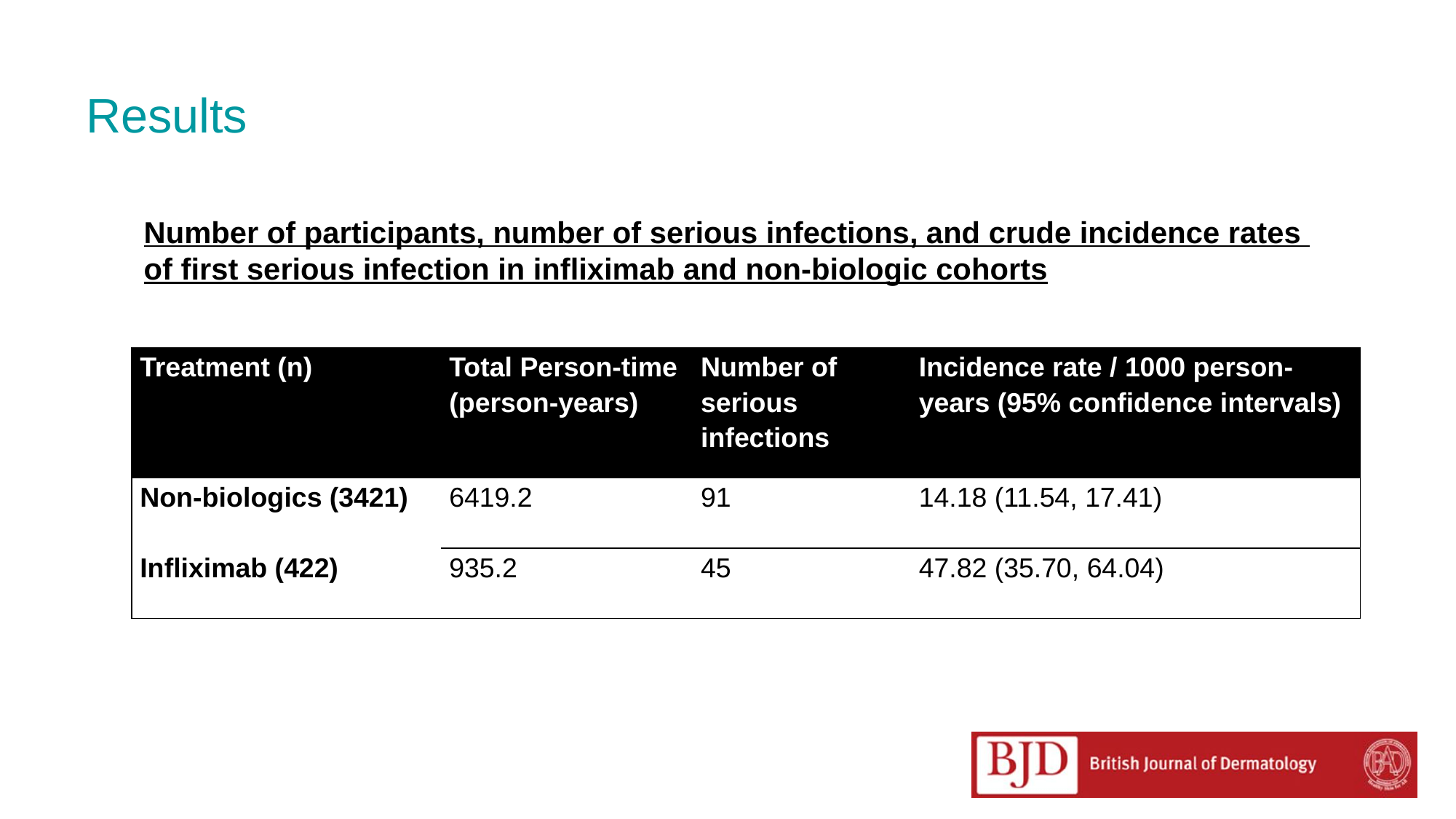

# Results
Number of participants, number of serious infections, and crude incidence rates
of first serious infection in infliximab and non-biologic cohorts
| Treatment (n) | Total Person-time (person-years) | Number of serious infections | Incidence rate / 1000 person-years (95% confidence intervals) |
| --- | --- | --- | --- |
| Non-biologics (3421) | 6419.2 | 91 | 14.18 (11.54, 17.41) |
| Infliximab (422) | 935.2 | 45 | 47.82 (35.70, 64.04) |

## Slide 8
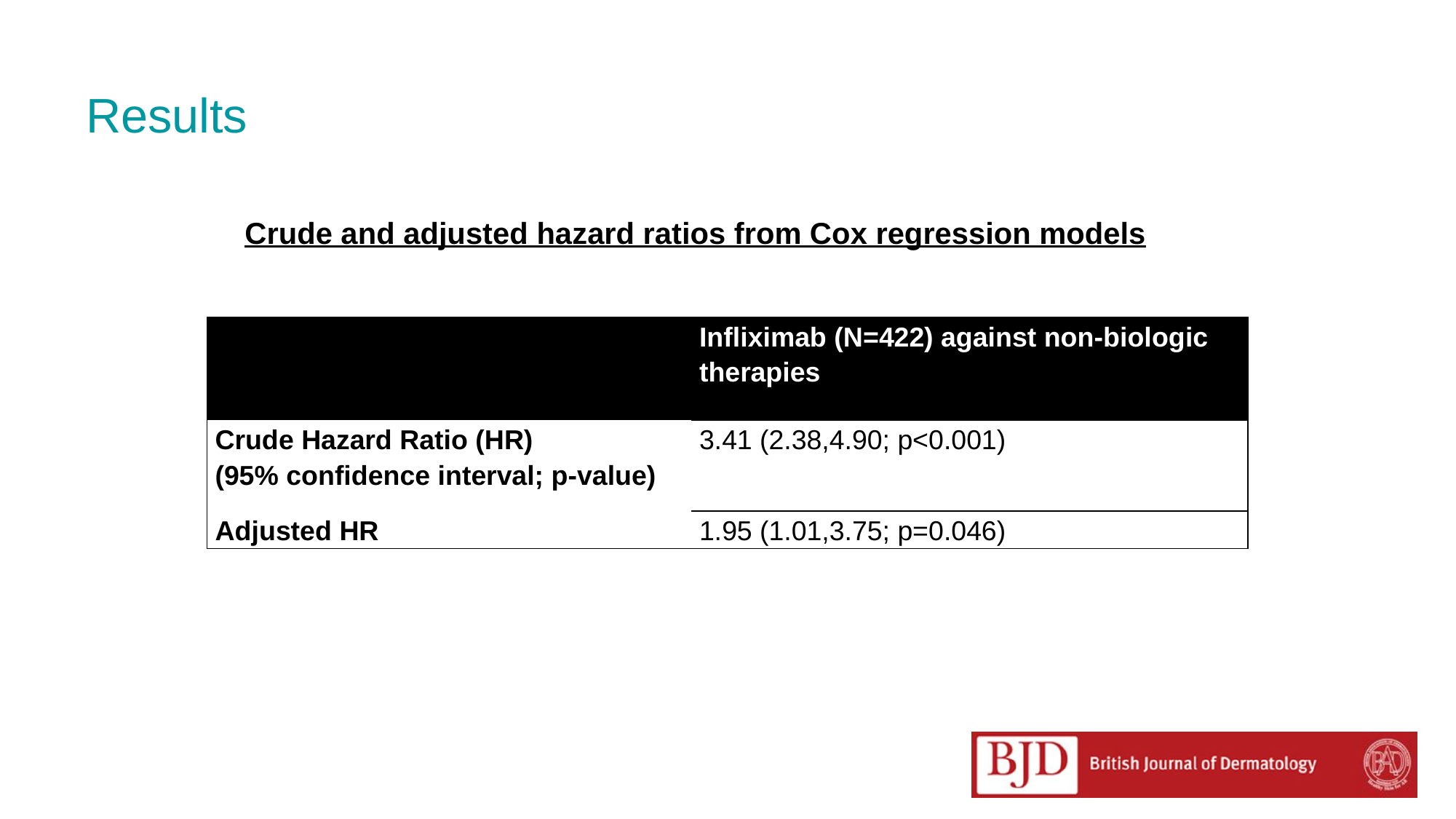

# Results
Crude and adjusted hazard ratios from Cox regression models
| | Infliximab (N=422) against non-biologic therapies |
| --- | --- |
| Crude Hazard Ratio (HR) (95% confidence interval; p-value) | 3.41 (2.38,4.90; p<0.001) |
| Adjusted HR | 1.95 (1.01,3.75; p=0.046) |

## Slide 9
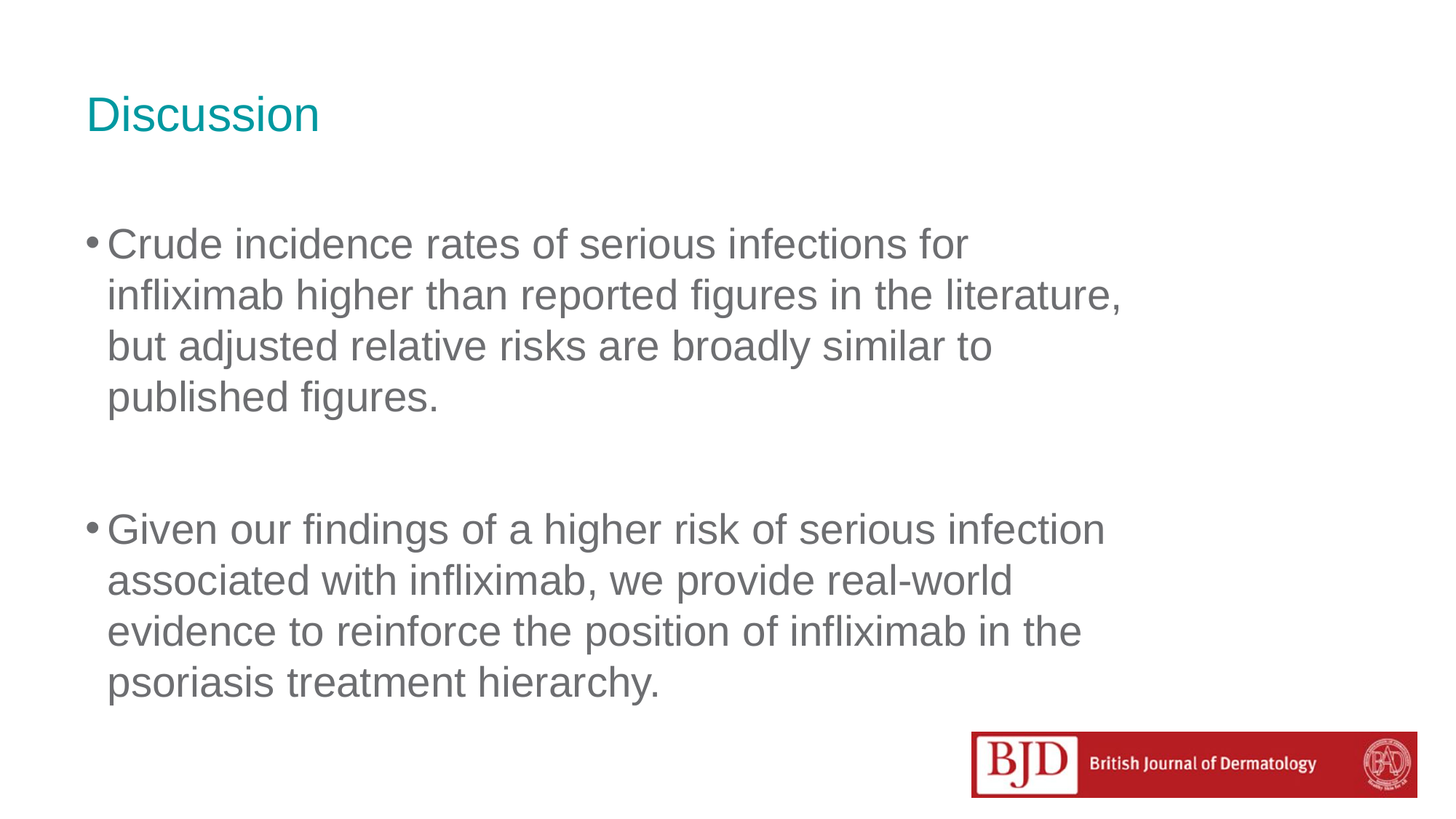

# Discussion
Crude incidence rates of serious infections for infliximab higher than reported figures in the literature, but adjusted relative risks are broadly similar to published figures.
Given our findings of a higher risk of serious infection associated with infliximab, we provide real-world evidence to reinforce the position of infliximab in the psoriasis treatment hierarchy.

## Slide 10
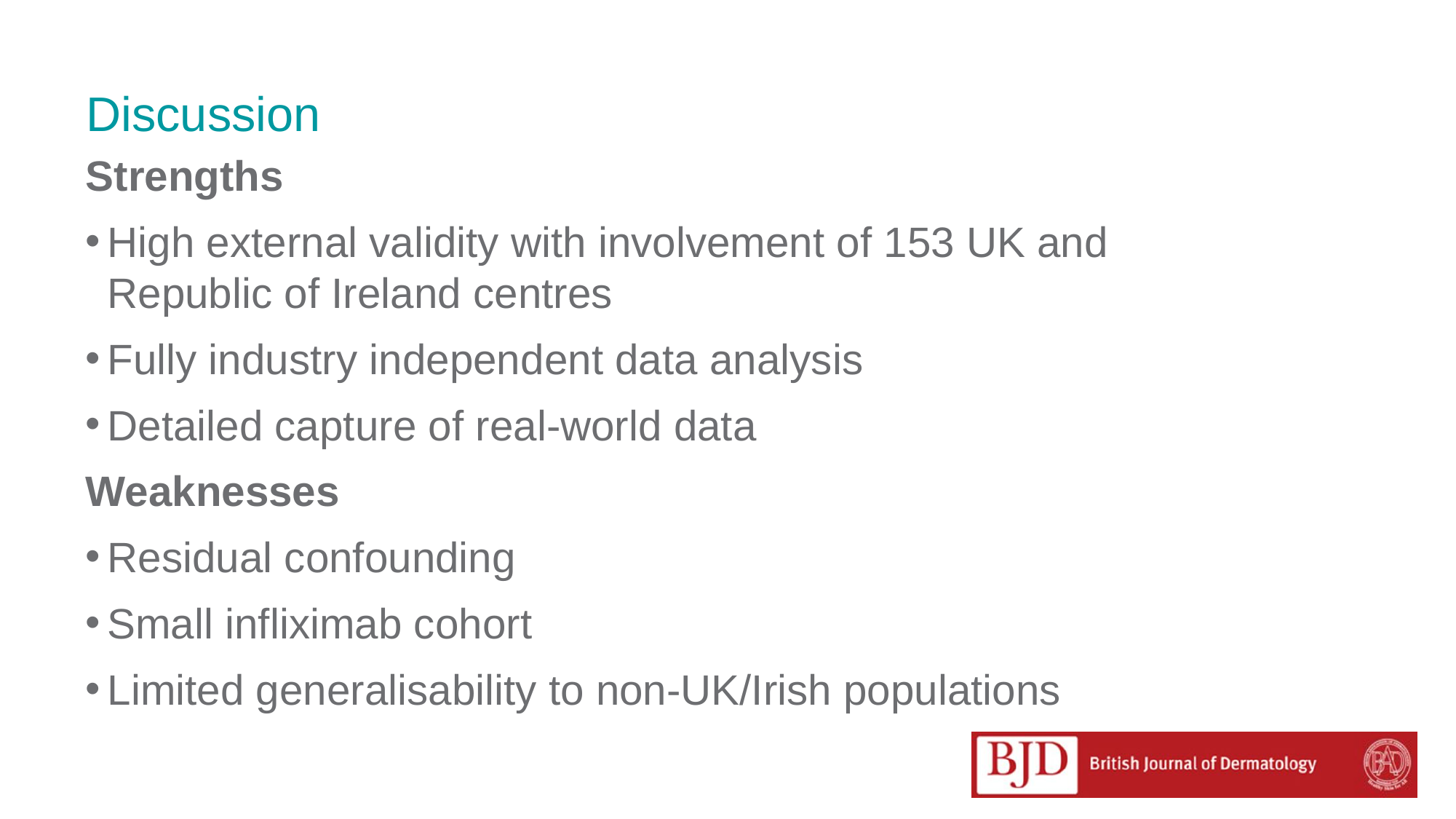

# Discussion
Strengths
High external validity with involvement of 153 UK and Republic of Ireland centres
Fully industry independent data analysis
Detailed capture of real-world data
Weaknesses
Residual confounding
Small infliximab cohort
Limited generalisability to non-UK/Irish populations

## Slide 11
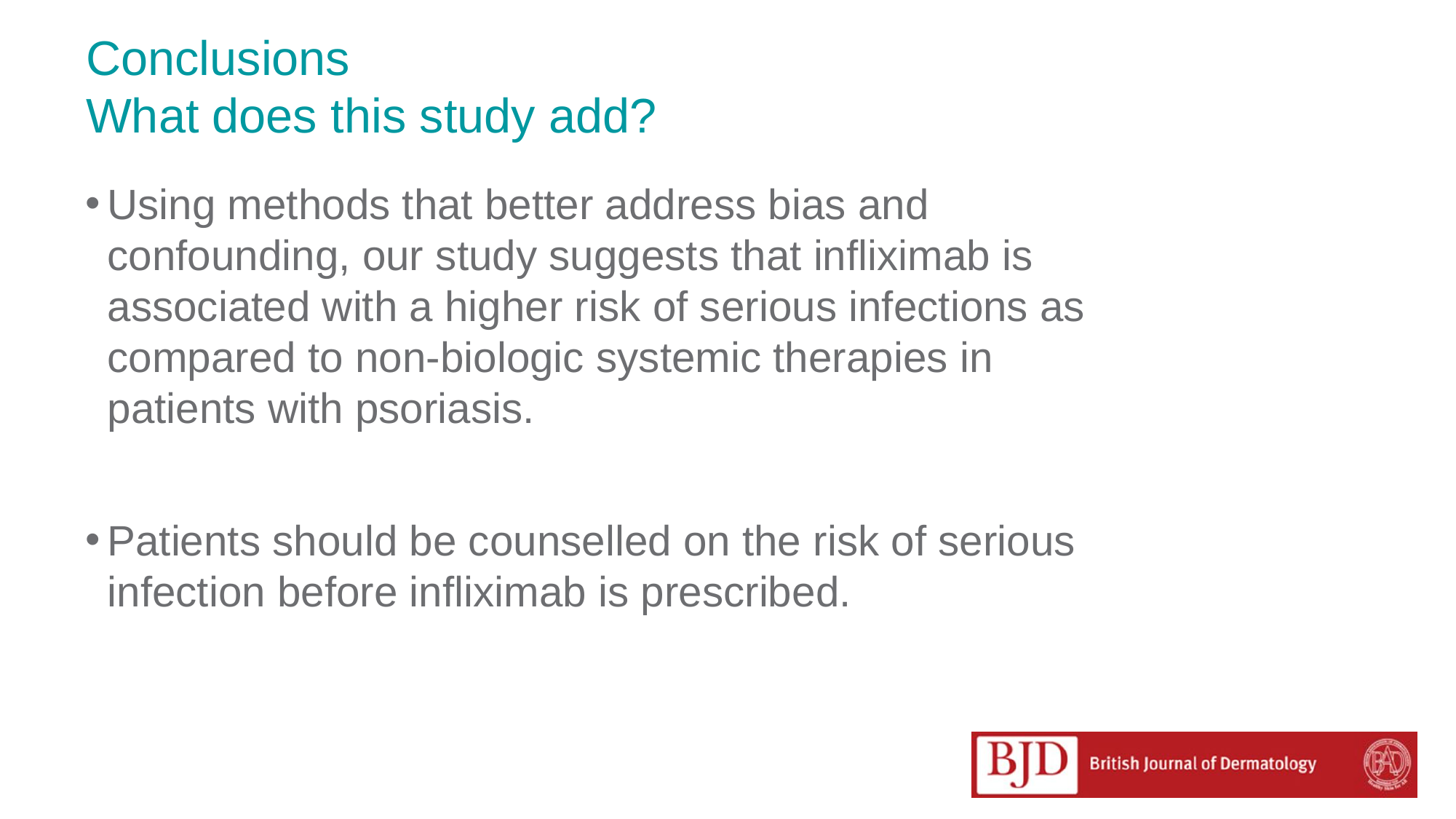

# Conclusions What does this study add?
Using methods that better address bias and confounding, our study suggests that infliximab is associated with a higher risk of serious infections as compared to non-biologic systemic therapies in patients with psoriasis.
Patients should be counselled on the risk of serious infection before infliximab is prescribed.

## Slide 12
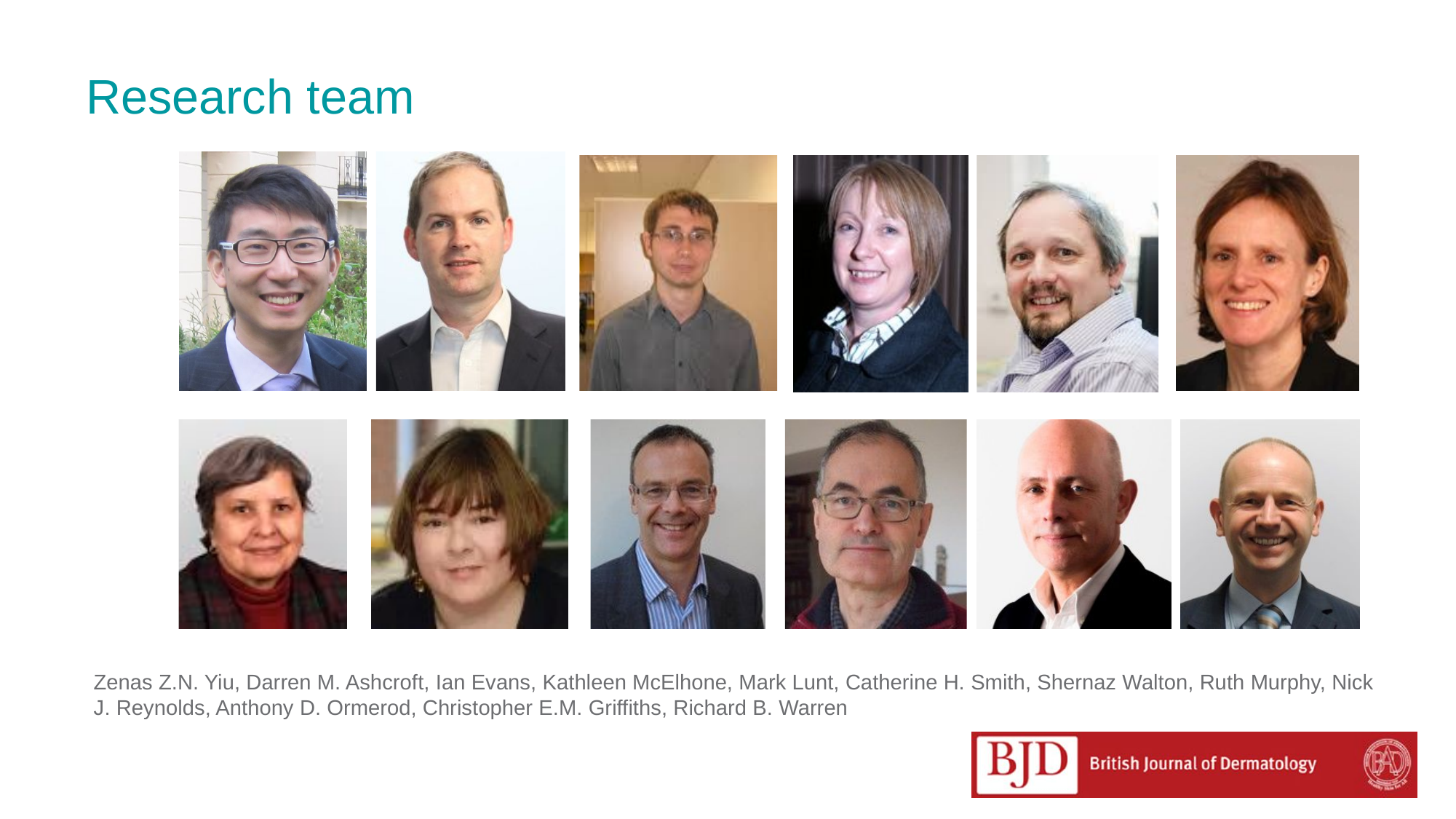

# Research team
Zenas Z.N. Yiu, Darren M. Ashcroft, Ian Evans, Kathleen McElhone, Mark Lunt, Catherine H. Smith, Shernaz Walton, Ruth Murphy, Nick J. Reynolds, Anthony D. Ormerod, Christopher E.M. Griffiths, Richard B. Warren

## Slide 13
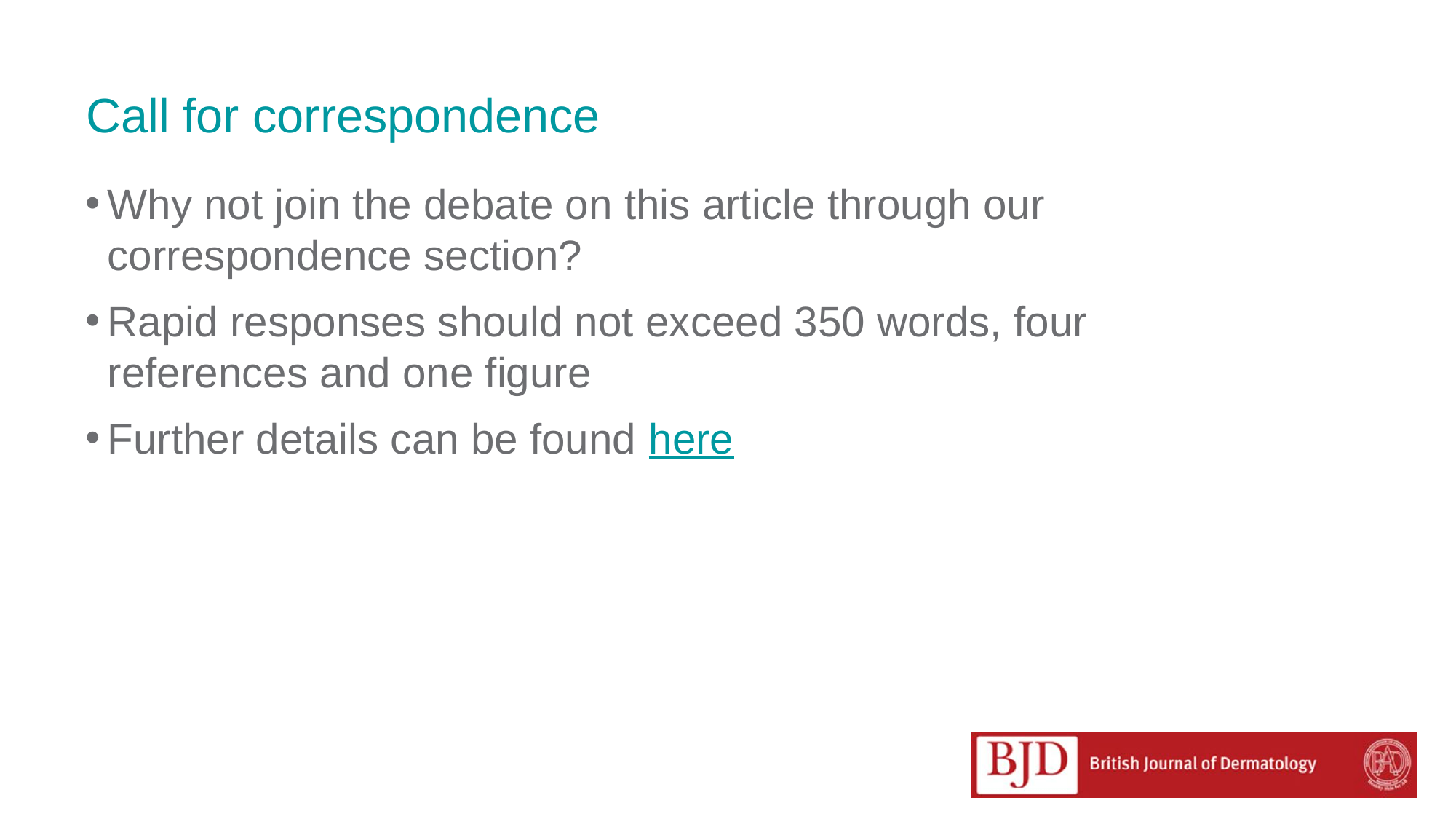

# Call for correspondence
Why not join the debate on this article through our correspondence section?
Rapid responses should not exceed 350 words, four references and one figure
Further details can be found here
